# Cryogels with controllable physico-chemical properties as advanced delivery systems for biomedical applications

**DOI:** 10.1016/j.mtbio.2025.101815

**Published:** 2025-04-29

**Authors:** Mucong Li, Xuyan Wei, Jiaqian You, Jian Feng, Xiuyu Liu, Jing Zhou, Qianju Wu, Yidi Zhang, Yanmin Zhou

**Affiliations:** a Hospital of Stomatology, Jilin University, Changchun, 130021, China; b Jilin Provincial Key Laboratory of Tooth Development and Bone Remodeling, Hospital of Stomatology, Jilin University, Changchun, 130021, China; c Stomatological Hospital of Xiamen Medical College, Xiamen Key Laboratory of Stomatological Disease Diagnosis and Treatment, Xiamen, 361000, China

**Keywords:** Cryogels, Drug and cell delivery, Injectability, Controlled release, Cell protection

## Abstract

Enhancing the spatiotemporal control of drug delivery holds significant potential for improving drug bioavailability, minimizing adverse reactions and toxicity, and advancing precision medicine. Traditional hydrogels have been widely recognized for their significance in substance delivery, attributed to their outstanding biocompatibility. Nevertheless, they exhibit certain limitations in the areas of controlled release and cell delivery. Cryogels, prepared using freezing technology, exhibit a unique macropores structure. The variations in technical parameters, such as the concentration of the gel precursor solution, freezing temperature, and freezing time facilitate adjustable mesh sizes and high pore connectivity. These characteristics are posited to enhance cell migration into the hydrogel interior and promote the transport of nutrients and metabolic waste. Their excellent toughness and injectability facilitate stress dispersion and provide effective cell protection. Consequently, cryogels modify drug release dynamics via their unique structure and integrate multiple release control mechanisms, thereby achieving spatiotemporal controlled delivery. This review paper provides an overview on synthesis methods, features, delivery advantages and mechanisms of cryogels. It further emphasizes the latest research advancements and applications of cryogels in spatiotemporal controlled delivery while acknowledging limitations associated with existing application methods.

## 1 Introduction

Drug delivery systems (DDS) comprise a range of effective carriers that transport pharmaceuticals to specific anatomical sites or tissues within a defined temporal window. With the evolution of medical technology, the demands placed on DDS have grown more rigorous. DDS must prevent the abrupt release of encapsulated drugs and molecules, ensuring instead a sustained and controlled release. This targeted delivery aims to enhance therapeutic efficacy, precision, and efficiency, while simultaneously reducing toxicity and adverse reactions [1,2]. Currently, various types of polymer delivery systems rely on four control modes: diffusion-controlled, swelling-controlled, erosion-controlled, and stimulation-controlled to deliver different types of drugs or molecules into the human body [3].

It is widely acknowledged that traditional Hydrogels consist of synthetic(e.g., PEG, PHEMA) or naturally derived (e.g., gelatin, alginate, HA) materials can form 3D networks after being cross-linked physically or chemically [4]. They can serve as a versatile drug or cell delivery carrier, facilitating interactions with cells in diverse physiological processes (e.g., diffusion, proliferation, migration, stem cell formation, differentiation) and pathological events (e.g., apoptosis, fibrosis, immune rejection) [5]. In terms of drug delivery, hydrogels are capable of regulating drug release via encapsulation. The rate of drug release is primarily determined by the degradation kinetics of the hydrogel, conventional biodegradable hydrogels cannot circumvent the initial burst release of drugs due to their accelerated degradation rate. Therefore, to achieve fully controllable spatiotemporal drug delivery, it is essential not only to regulate degradation but also to integrate diffusion regulation, swelling regulation, stimulus response, and affinity modulation. In terms of cell delivery, traditional hydrogels exhibit excellent biocompatibility and effectively mimic the intricate physical and biochemical properties of the natural extracellular matrix (ECM). Consequently, they are widely utilized for cell embedding in cell culture systems and delivery applications [6]. However, the small pore sizes (usually in the nanometer range) and insufficient pore interconnectivity of conventional hydrogels constrain cellular activities essential for biomedical applications, such as cell motility, spreading, and the diffusion of proteins, oxygen, and nutrients/waste products, and it is impossible to load cells into the pores, thereby limiting their utility [7,8].

Cryogels, prepared via freezing processes, boast superior structural and property benefits over conventional hydrogels. They effectively facilitates the controlled release of loaded substances as smart DDS [9]. In the field of controlled delivery, the application advantages of Cryogels are primarily ascribed to the structural transformations induced by freezing and the modulation of drug release mechanisms. In the degradable system,the freezing process leads to the concentration of the gel precursor solution, thereby enhancing the cross-linking strength of cryogels and altering their swelling and degradation behaviors. Additionally, the large, interconnected porous structure modifies the release kinetics of encapsulated drugs, enabling them to achieve equilibrium within the network before being gradually released into the surrounding environment [10]. In terms of cell delivery, the larger pore size of cryogels facilitates cellular adhesion while promoting their infiltration into the mesh, thereby enhancing the rate of cell loading. Moreover, the unique structure formed through freezing confers high elasticity and exceptional shape memory properties to cryogels and renders them suitable for injectable applications [11]. The deformation during the injection process serves to mitigate stress, thus facilitating cell delivery while simultaneously ensuring a high rate of survival and optimal biological functionality [12]. Besides, achieving injectability in drug and cell delivery reduces the technical demands on surgeons and diminishes surgical trauma and patient discomfort throughout the diagnostic and therapeutic procedures. It also prevents postoperative infections, scarring, and potential complications in both hard and soft tissues associated with surgical incisions [13,14] and avoids the catastrophic impact on the transplanted cells/tissues or materials the greatest extent [15]. Moreover, the synthesis process of cryogels is straightforward and does not necessitate specific materials and the physical and chemical properties of cryogels, including pore size, porosity, interpore connectivity, hardness, etc., can be fine-tuned by adjusting the precursor gel concentration and freezing parameters. This versatility makes cryogels suitable for various applications in controlled substance delivery. This paper first elaborates on the synthesis process of Cryogels, systematically compares the similarities and differences between Cryogels and traditional Hydrogels, and subsequently highlights the advantages of Cryogels as a material delivery system. It reviews the application advancements and operation method of Cryogels in the field of intelligent spatiotemporal controlled delivery and their clinical translation in disease treatment. Additionally, the limitations, potential improvement strategies, and future development prospects of Cryogels are thoroughly discussed.

## 2 Characteristics and application advantages of cryogels

### 2.1 The preparation of cryogels

**Fig. 1:**
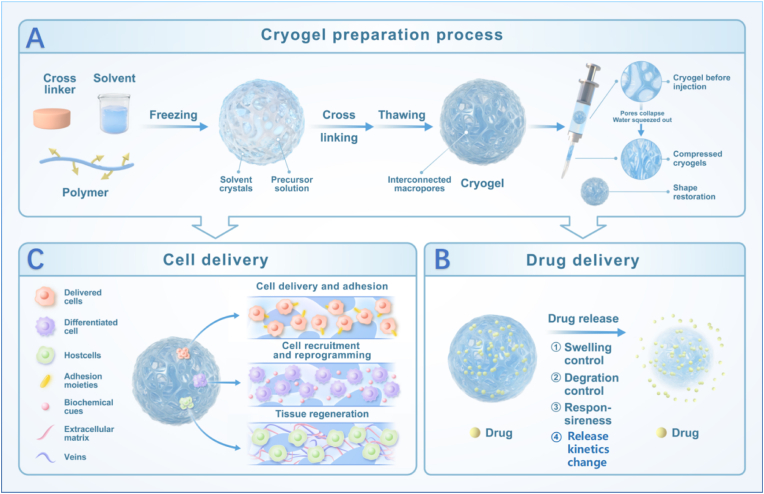
Graphic abstract. (A) The preparation process for cryogels is demonstrated along with their injectable properties. (B) The applications of cryogels as delivery vehicles and their principles (C) The role of cryogels in cell delivery.

Cryogels are a special type of Hydrogel formed at subzero temperature. The synthesis of cryogels involves a series of frozen steps resulting in macroporous interconnected network. The formation process of cryogels is presented in Fig. 1A.

Below is a detailed description of the synthesis process [16].

● Preparation of precursor solution: a monomer (e.g., acrylamide [[17], [18], [19], [20]], gelatin [15,[21], [22], [23]], chitosan [[24], [25], [26], [27], [28], [29]]) and a crosslinker (e.g., N, N′-methylenebisacrylamide [[30], [31], [32]], glutaraldehyde [33]) are dissolved in an aqueous solvent (or organic solvent for certain systems). Initiators (e.g., ammonium persulfate, TEMED) or catalysts may be added to induce polymerization.
● Freezing: the homogeneous solution is subjected to controlled freezing. During freezing, ice crystals nucleate and grow, forming a network of solvent crystals while concentrating the monomers/crosslinkers in the interstitial regions. As a natural porogen, ice crystals not only eliminate the potential biological toxicity associated with residual organic porogens but also enhance the interconnectivity rate among pores, addressing the limitations of the sacrificial particle method [34].
● Polymerization/Crosslinking: polymerization occurs in the unfrozen liquid microphase surrounding ice crystals, creating a crosslinked polymer network. The reaction time varies (hours to days) depending on the system (e.g., free-radical polymerization for synthetic cryogels or Schiff-base reactions for biopolymer-based cryogels).
● Thawing: the frozen system is slowly warmed to room temperature, allowing ice crystals to melt and leaving behind interconnected macropores (10–200 μm). The resulting cryogel retains elasticity and high permeability due to its porous structure [11].

### 2.2 Parameter regulation of cryogels preparation process

**Fig. 2:**
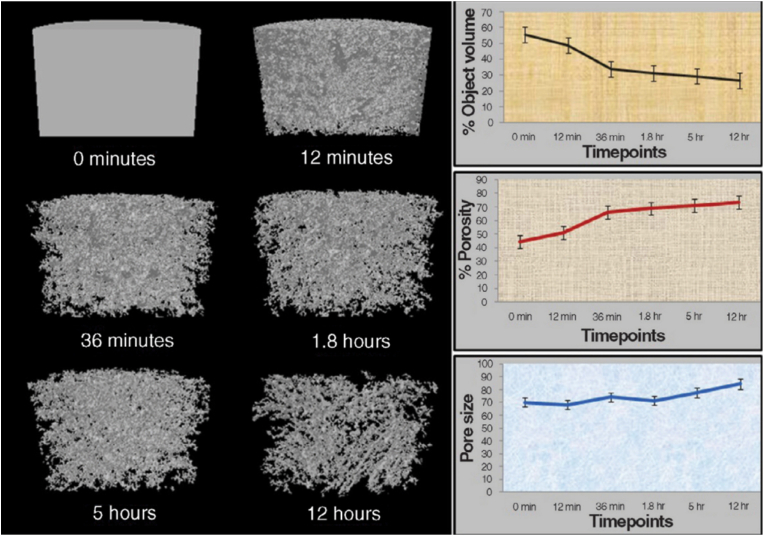
Variation of volume, porosity, and average pore size of Polyacrylamide cryogels with freezing time; At 0 min, ice crystal formation was not initiated, and the Polyacrylamide solution achieved homogeneity. As the freezing time progressed, crystal nuclei gradually emerged and ice crystals continued to grow. The black region represents solvent crystals, while the gray region depicts the Polyacrylamide solution undergoing crystallization without any ice crystals. Represented with the permission from Ashok Kumar et al. (2010), Materials Today [35].

**Fig. 3:**
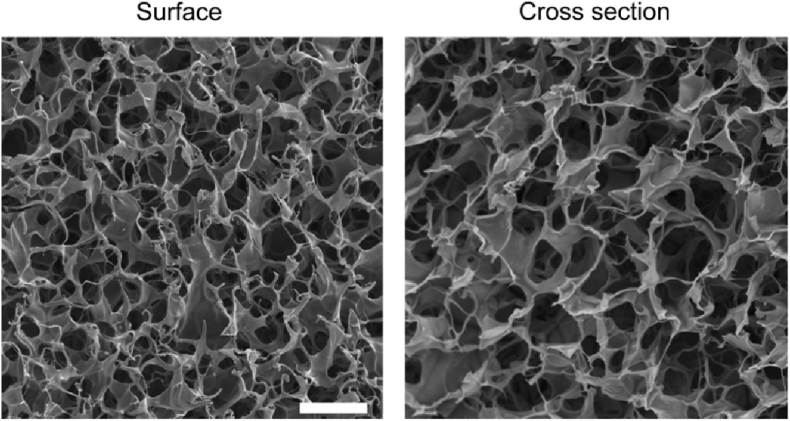
The pore size of the cryogels progressively increases from the surface to the interior, displaying a depth-dependent variation. (scale bar = 50 μm). Represented with the permission from Koshy et al. (2014), Biomaterials [15].

Freezing is a crucial step in the preparation of cryogels. The porosity and mesh size of cryogels can be modulated by adjusting the freezing time during their fabrication (Fig. 2). According to the mesh formation process of cryogels described earlier, extending freezing time below the solvent freezing point temperature can cause further formation of ice crystals and increase the mesh size after temperature recovery [35]. The rate of cooling significantly influences the process of ice crystal formation by determining the speed and pattern of crystallization. Rapidly lowering the temperature or decreasing it excessively during the freezing process can impede further aggregation of ice crystals and may result in the premature cessation of ice crystal agglutination. Finally, cryogels with small porosity, mesh size, and interpore connectivity have been obtained [9,[36], [37], [38]]. The freezing temperature during cryogel preparation is inversely proportional to the mesh size of the resulting cryogels; specifically, a lower freezing temperature leads to a smaller mesh size. Ivanov et al. prepared polyacrylamide (PAAm) cryogels by lowering the freezing temperature by 15 °C and reducing the pore size of the cryogels by 30 μm on average [39]. The dimensions of cryogels can influence the mesh size distribution across regions. Notably, in the fabrication of large cryogels, variations in the freezing rate and time between the surface and core reaching the set temperature result in differential ice crystal aggregation, leading to distinct mesh sizes and porosities between the surface and core of the final product. This phenomenon has been confirmed by Koshy's research (Fig. 3) [15]. While this feature is considered a disadvantage in the design and application of traditional support materials and delivery carriers [40], it also offers novel insights into the design of anisotropic support materials as declared by Sen et al. in their research due to the heterogeneous morphology naturally present in human tissue [41].

### 2.3 Comparison of cryogels with hydrogels

Due to their unique preparation process, cryogels exhibit significant differences compared to traditional hydrogels in various physical properties and application methods, as illustrated in Table 1.

**Table 1:** Comparison of cryogels and hydrogels.

	Cryogel	Corresponding hydrogel
Pore/Mesh size	interconnected macropores in the range of 1–10^2^ μm [42,43]	nanometer range pores which were smaller, less connected, and poorly distributed [11]
Interconnection rate	Highly connected mesh [44]	poorly connected pores
Cross-link density	Higher; (freezing progress causes concentration of the precursor solution)	Lower
mechanical property	Due to their high porosity, the stiffness of cryogels decreases, however, this also results in increases in toughness and elasticity [16,40,[45], [46], [47], [48], [49]]. Cryogels can undergo reversible compression at strain levels exceeding 80 % [42]. This shape memory property is the basis for its injectable properties.	lower elasticity and enhanced toughness; brittleness increases after absorbing water.
Swelling	faster [33]	slower
Cell loading	a. Loading on the surface b. Loading in the Interconnected mesh [21,22,[50], [51], [52], [53], [54], [55], [56], [57]]	a. Loading on the surface b. Cell embedding/in situ gelation [[58], [59], [60], [61]]

## 3 Cryogels as vehicles for cotrolled spatiotemporal delivery of bioactive molecules and cells

With the advancement of research, cryogels have emerged as versatile drug delivery carriers that encompass not only synthetic monomers but also natural polymers such as water-soluble Kefiran polysaccharides [62]. Furthermore, their application extends beyond blocks and spheres to include micelles [63] and microneedles [64]. These cryogels can effectively deliver a wide range of active ingredients, including both drugs, chemical fertilizers [65], peptides [66], proteins [10,[67], [68], [69], [70]], etc. [71].

### 3.1 The distinctive advantages of cryogels as sustained-release systems

**Fig. 4:**
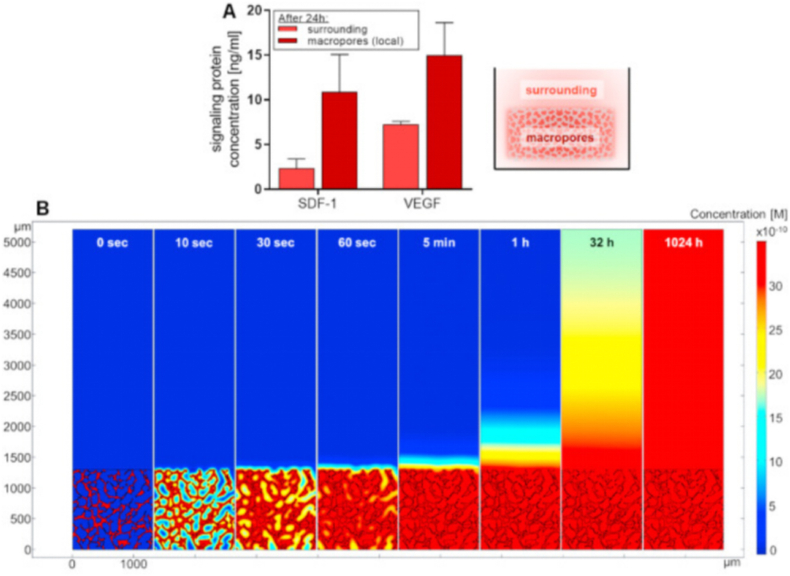
Influence of the macroporous of starPEG-heparin cryogels on signaling protein release kinetics: A) Volume concentration of the signaling protein in the surrounding medium and locally within the macropores of the cryogels after 24 h. B) Theoretical mathematical modeling of VEGF release from a 2D representation of the starPEG-heparin cryogel (3.9 mm heparin) based on a reaction-diffusion model. The color scale indicates the VEGF concentration. At concentrations of 30 × 10^−10^ M or higher, the free factor is represented by the same color (dark red). Represented with the permission from Sievers et al. (2021), Biomaterials [10]. (For interpretation of the references to color in this figure legend, the reader is referred to the Web version of this article.)

Compared with traditional hydrogels, the most significant advantage of cryogels as a sustained-release system lies in their macroporous structures, which play a critical role in determining release kinetics. Specifically, drug or signaling molecules encapsulated within cryogels first establish concentration equilibrium within the macropores and are then released into the surrounding medium, driven by both the concentration gradient and solution flow dynamics [10]. The study by Sievers et al. represents the first systematic explanation of this phenomenon: they performed experiments and simulated VEGF release in the polymer network of starPEG-Heparin Cryogel Strut using a reaction-diffusion model [72,73]. The simulation results were in excellent agreement with the experimental data, demonstrating that VEGF concentrations inside the macropores of the cryogels reached equilibrium more rapidly than around the cryogels (Fig. 4). In a selected 2D model of starPEG-Heparin cryogel derived from cross-sectional confocal microscope images, equilibrium concentration of VEGF in macropores were reached within a few minutes. Conversely, due to the structural characteristics of the cryogel, most of its surface is oriented inward, and in the absence of convection, it takes approximately 40 days to reach a similar concentration in the surrounding medium (i.e., about 4 mm from the outer surface of the scaffold) to achieve a comparable concentration [10]. Secondly, in contrast to traditional hydrogels, the unique freezing process of cryogels induces alterations in swelling rate, swelling speed, crosslinking degree, and degradation rate. Furthermore, it can influence the release of drugs or molecules to a certain extent, necessitating analysis in conjunction with the specific gel structure and molecular characteristics. Specifically, the study of Almeida et al. reports the development of chitosan cryogels (CS) capable of releasing NO triggered by the rapid hydration process [74]. The rapid swelling property of cryogels leads to the responsive release of NO. Goodarzi and colleagues utilized GelMA as the gelator matrix for cryogel fabrication, and incorporated cellulose nanocrystal (CNC) and PAMAM nanomaterials to enhance their mechanical properties. Subsequently, they investigated the sustained release effect of these composite cryogels on the anti-inflammatory corticosteroid drug betamethasone sodium phosphate (BSP). The findings demonstrated that compared to cryogels synthesized solely with GelMA, the mesh size of the composite Cryogels decreased while the Young's modulus increased from 4 kPa to 14 kPa. Moreover, a drug release assay revealed that the nanoparticle (NP) modified cryogel effectively prolonged BSP release compared to the GelMA cryogels. However, this effect was observed only within a duration of 24 h [75], necessitating further exploration and endeavors to enhance the sustained-release capability of cryogels. Relying on the unique physical properties and release kinetics of cryogels compared to hydrogels, it is feasible to modulate drug release to a certain extent. By integrating the characteristics of cryogels with various responsive mechanisms commonly applicable to hydrogel systems, more intelligent and controllable material delivery can be achieved. The subsequent sections will elaborate on these concepts in detail.

### 3.2 Affinity regulation

**Fig. 5:**
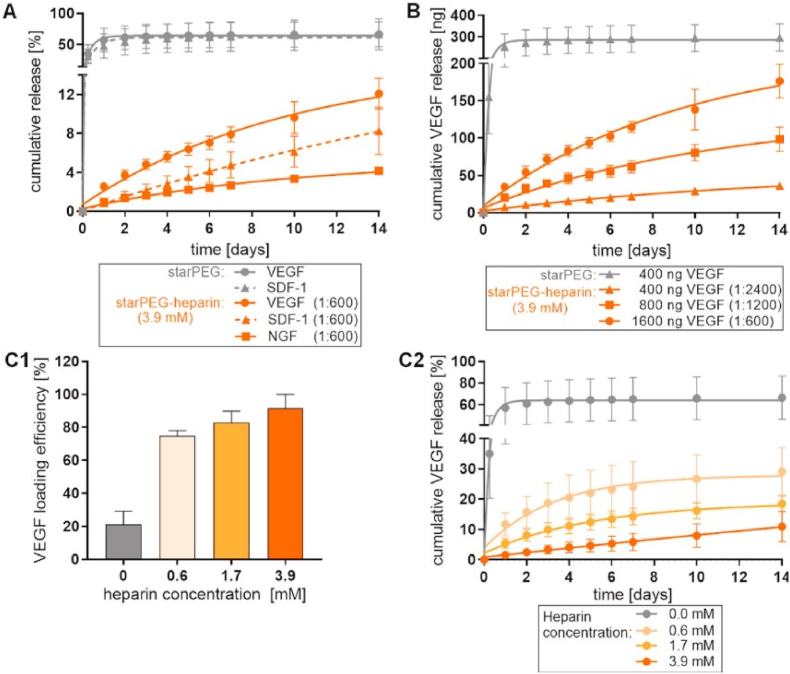
The release curve (from starPEG-heparin cryogels) of signaling protein and its influencing factors. A: The release curves of VEGF, SDF-1, and NGF at the same concentration (from starPEG-heparin (3.9 mM heparin) and starPEG cryogels). B: Release curves of different VEGF loading concentrations (from starPEG-heparin cryogels (3.9 mM heparin) and starPEG cryogels). C1: The influence of heparin concentration on the loading efficiency of VEGF (1000 ng in the loading solution). C2: The influence of heparin concentration on the VEGF release curve. Represented with the permission from Sievers et al. (2021), Biomaterials [10].

**Fig. 6:**
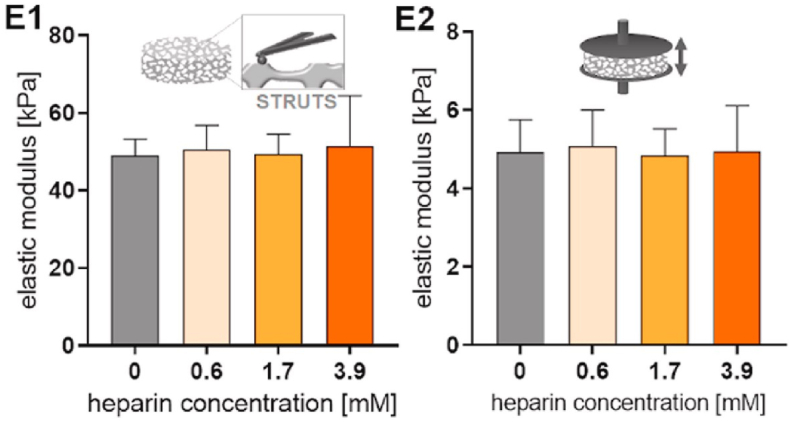
E1: The local and E2: global elastic moduli of starPEG-heparin at different heparin concentrations. Represented with the permission from Sievers et al. (2021), Biomaterials [10].

For an extended period, drug release has primarily been controlled through modulating diffusion resistance and regulating the degradation rate of the delivery system. However, only a limited number of studies have explored affinity-based drug delivery cryogel systems, which integrate synthetic charged building blocks, additives, or highly charged natural polyelectrolytes such as heparin or chitosan to mitigate initial burst release and extend release kinetics [10,67,70,[76], [77], [78], [79]]. The interactions between drugs and gels, as well as among gel components, can be mediated through covalent cross-linking or physical binding, the specific binding mode also exerts a certain influence on the drug release rate, as demonstrated by the study of Mehrez et al. They developed an environmentally friendly cryogels using natural polymers hydroxyethyl cellulose (HEC) and bacterial cellulose (BC), incorporated Ag@TiO_2_ NPs as an active agent to enhance the antibacterial activity of the system, and encapsulated the pesticide Tebuconazole within cryogels for controlled release. It was observed that varying proportions of Ag@TiO_2_ NPs altered the interactions between the drug and the carrier system (e.g., hydrogen bonding, van der Waals forces, hydrophobic interactions), resulting in distinct release profiles [80]. Similarly, Koshy and colleagues integrated various protein factors with Laponite nanoparticles into alginate cryogel. by leveraging electrostatic affinity regulation, the release kinetics of the protein were modulated, leading to a sustained-release profile [67]. Kim et al. engineered a heparin-functionalized gelatin cryogel as a controlled-release carrier for VEGF. Owing to the affinity-based regulation of heparin-binding proteins on VEGF molecules, the heparin-gelatin cryogel demonstrated superior control over the release kinetics of VEGF [77]. However, the above studies have also modified the mechanical properties, swelling rate, and other physical characteristics of cryogels while achieving affinity regulation. Ideally, the functionalization process should not alter the physicochemical properties of the cryogels system while modulating the affinity between loaded proteins and gel matrix. The work by Sievers et al. addresses this challenge. Their development of macroporous starPEG-heparin cryogels enables the regulation of protein factor release via affinity and electrostatic interactions. Furthermore, the loading and release kinetics of protein factors can be fine-tuned by adjusting the amount of heparin incorporated and protein factors. The charges carried by different types of protein factors vary, thus showing different affinities for cryogels and different release curves (Fig. 5). Notably, this approach does not change the local or global mechanical properties of the cryogel system (Fig. 6), thereby enhancing the controllability and reliability of cryogel performance [10].

### 3.3 Enzyme responsiveness

For degradable cryogels, the degradation of the delivery system is an inevitable process, and it is also an important step in regulating the release rate. By changing the number of enzyme action sites in the cryogel matrix or recruiting specific hydrolases, the drug release process can be regulated by controlling the degradation rate of the cryogels. Lozinsky et al. developed wide-porous protein-based cryogels derived from bovine serum albumin for the controlled release of dioxidine. Succinylation of these cryogels not only enhanced their swelling but also diminished the number of trypsin enzymatic reaction sites, thereby improving the cryogels' resistance to enzymatic degradation [81]. Additionally, Koshy incorporated neutrophil-macrophage colony-stimulating factor into the precursor gel solution. This factor was released progressively as the cryogel degraded, recruiting immune cells to infiltrate the scaffold and stimulating the production of metallo-matrix proteases, further expediting the degradation process of cryogels to achieve controlled and enzyme responsive release [15].

### 3.4 Stimulus-responsive cryogels facilitate spatiotemporal regulation

In the treatment of numerous diseases, targeted drug or cell delivery has emerged as a pivotal approach to mitigate adverse reactions and optimize therapeutic efficacy. Disease-induced alterations in the local microenvironment, such as variations in temperature and pH, present a compelling opportunity for targeted drug delivery strategies. These include dynamic pH variations within the gastrointestinal tract, acidic conditions at inflammation sites, and elevated temperature environments. For the control of drug delivery, the present research encompasses both the refinement of drug delivery systems to modulate release kinetics and ensure precise control over drug release, as well as the development of stimulus-responsive systems capable of triggering drug release under defined conditions or within specific locales through variations in stimuli like temperature and pH [[82], [83], [84], [85]]. These alterations in stimulus conditions will induce structural and functional changes within the delivery system. Herein, specific descriptions are provided using examples of temperature-responsive cryogels, pH-responsive cryogels, and temperature/pH dual-responsive cryogels.

#### 3.4.1 Temperature responsiveness cryogels

Previous studies have claimed that the rate of drug release is primarily influenced by two factors, i.e., the drug diffusion rate and the solubilization of the drug delivery system during the delivery process. The temperature-responsive cryogels undergo changes in intra- and intermolecular hydrogen bonding within the gel system at different temperatures, resulting in alterations to the hydration state and volume of the cryogels during operation. These variations are ultimately manifested as changes in the swelling ratio, which leads to a denser hydrogel system with decreased mesh size and consequently causes a decelerated release of drugs encapsulated within the system [86]. Thermosensitive cryogels can be classified as positive or negative temperature-sensitive systems. A positive temperature-sensitive cryogels has an upper critical solution temperature (UCST). Such cryogels contract upon cooling below the UCST. Negative temperature-sensitive cryogels have a lower critical solution temperature (LCST). These cryogels contract upon heating above the LCST [87,88]. In order to investigate the differences in structure between the hybrid cryogels and ordinary CNF cryogels at the same temperature, and the changes in structure with temperature, Zhang et al. prepared poly-NIPAm modified cellulose nanofibril cryogels by incorporating temperature-sensitive monomer NIPAm (N-isopropylacrylamide) into the cellulose nanofiber (CNF) cryogel microbeads. They verified the effect of this structural change on drug release by loading 5-fluorouracil (5-FU) into them. PNIPAm is a well-known thermoresponsive polymer, with a lower critical solution temperature (LCST) of around 32–33 °C [[89], [90], [91]]. The results showed that when the temperature was raised from 22 °C to 36 °C, the volume of the CNF-PNIPAm cryogels decreased. This change extended the initial burst release of equal 5-FU quantities from seconds to an hour, transitioning to a gradual release phase. Within 60 min at 22 °C, the cumulative 5-FU release was 89 % for AM-1(NIPAm accounts for 6 % (w/v) of CNF-PNIPAm) and 70 % for AM-4(NIPAm accounts for 15 % (w/v) of CNF-PNIPAm), whereas at 37 °C, it was 66 % and 44 %, respectively [92]. The temperature-responsive cryogels were employed to mitigate the initial burst release, thereby achieving a more controlled and gradual drug delivery profile. The study conducted by Bistra et al. reached the same conclusion that, unlike conventional hydrophilic gel carriers, poly (ethoxytriethylene glycol acrylate) cryogels exhibit hydrophobic properties and a reduced swelling ratio at temperatures above the lower critical solution temperature (LCST). This unique characteristic facilitates sustained release of verapamil for a minimum duration of 8 h or more [93]. Cryogels synthesized from other materials according to this method share similar properties [94,95]. However, substantial scope remains for the further development and refinement of temperature-responsive cryogels. For example, in Larsson's research, his CNC-enhanced PNIPAAm cryogels demonstrated a decrease in solubility ratio and volume collapse when heated above the metamorphic temperature. The reason behind this phenomenon lies in the increased stiffness of the pore walls within the cryogel, which restricts gel collapse from occurring. Even after an extended period of time, lowering the temperature below the phase transition point does not result in complete re-solubilization due to this collapse effect [89]. This suggests that despite cryogels' demonstrated potential in drug release control, the optimization of their specific performance is still a key research priority. To solve this problem, Deng et al. conducted a study and developed a cryogel with thermoresponsive pressure-dependent conductivity, which exhibited rapid thermal responsiveness and reduced the time for collapse re-solubilization from several weeks to just 2 min. Furthermore, this cryogel demonstrated enhanced reversibility of structural changes, enduring ten complete solubilization-desolubilization cycles [96]. In this study, besides temperature responsiveness, the magnetic responsiveness and pressure-dependent electrical conductivity rely on doping materials possessing relevant properties. Doping these materials or incorporating branched side chains not only endows cryogels with magnetic responsiveness but also brings about structural changes to the cryogels. Consequently, such modifications can either enhance or diminish the temperature responsiveness of cryogels. Therefore, modifying stimuli-responsive cryogels requires careful consideration to optimize their properties [97], when adjusting the proportion of a certain component in cryogels to change their responsiveness, it is best not to affect the other physicochemical properties of the cryogels.

#### 3.4.2 PH responsiveness cryogels

PH responsive cryogels primarily modulate the drug release rate by adjusting the extent of cryogel swelling. Environmental pH and ion concentration can alter the degree of ionization of specific functional groups within cryogels, thereby influencing the swelling behavior of these cryogels. The pH-induced variation in the swelling ratio and mesh size of cryogels modulates drug diffusion resistance. The variation trend of swelling rate of pH-responsive cryogels is primarily determined by the degree of ionization, and the pKa value of functional groups within cryogels. [98]. The classification of these compounds is based on their functional groups, which can be divided into three distinct categories: (1) polyacids containing weakly acidic functional groups, such as -COOH or -SO_3_H; (2) polybases containing weakly basic functional groups (-NH_2_); and (3) amphoteric ions possessing both weakly acidic and weakly basic functional groups [[98], [99], [100]]. The pH-sensitive plantain and polyacrylamide-based hydrogel, designed by Baljit et al., exhibited a collapsed polymerized state under low pH conditions due to the non-ionization of the -CONH_2_ group within it. Conversely, at high pH levels, partial ionization occurred, leading to repulsion between charged -COO^-^ groups and subsequent polymer swelling [99]. In addition, Wang et al. demonstrated that variations in the swelling rates of pH-responsive cryogels at different pH levels also influenced their degradation rates. Specifically, when the swelling ratio was lower, the degradation process occurred more slowly due to reduced hydrophilicity of the cryogels. These findings indicate that alterations in the swelling rate of cryogels induced by environmental pH can lead to secondary effects on degradation rates, thereby further regulating drug release [101]. The body's distinct regions possess unique microenvironments, marked by subtle differences in substance distribution and pH levels. This phenomenon is particularly evident within the digestive system, where orally administered drugs traverse through diverse pH environments, including a pH range of 6.2–7.3 in the oral cavity, a pH of 7 in the esophagus, a pH range of 1.5–3.5 in the stomach, a pH of 6 in the small intestine, a pH of 7.4 at the end of ileum, and finally, a range of pH from 5.7 to 6.1–7.5 in the colon [102]. Meanwhile, the colon harbors a substantial population of bacteria capable of secreting specific enzymes. This inherent pH gradient [99], coupled with the presence of these specialized enzymes, offers potential for targeted drug delivery to the colon and the site-specific degradation of carriers within this region [103]. Han et al. developed pH-responsive cryogels composed of carboxymethyl cellulose (CMC) and utilized Al^3+^ as a crosslinking agent for the colon-targeted delivery of resveratrol. The variation in the swelling behavior of the cryogels at different pH levels effectively inhibited the premature release of resveratrol in the stomach. Upon reaching the colon, the cryogels were enzymatically degraded by cellulase secreted from intestinal microorganisms, thereby ensuring the precise targeted release of the drug [104].

The pH-induced variation in the swelling ratio of cryogels is just one of several factors that can modulate drug release rates and cumulative amounts, rather than being the exclusive determinant. The drug's solubility, the cross-linking density of cryogel carriers, their sensitivity to pH-induced alterations, and the drug-carrier interactions under varying pH conditions all significantly contribute to the drug's release profile. The study conducted by Waria et al. examined the attachment of poorly water-soluble diclofenac sodium (DS) and indomethacin (IDM) to chitosan-plagioclase zeolite cryogels, investigating their release characteristics. The findings indicated that protonation of cryogels at pH 1.2 resulted in an enhanced swelling rate of the carriers compared to that at pH 7.4. Contrary to expectations of diminished drug diffusion resistance and enhanced release, the cumulative drug release was less than 5 %, with a slower rate at pH 1.2 but exceeding 70 % at pH 7.4. This discrepancy was attributed to DS's extremely low solubility and strong interaction with CS under acidic conditions, significantly reducing the release. At pH 7.4, the amine groups of cryogels are deprotonated, resulting in the disruption of the interaction between the drug and the cryogels matrix, as well as an enhancement in the drug's solubility. Despite a reduction in the swelling rate of cryogels, which increases the diffusion resistance of the drug, the overall drug release rate remains significantly higher than that at pH 1.2 [105]. Therefore, the responsiveness of the carrier represents a critical factor influencing the drug release rate, yet it is not the sole determinant. The extent of the drug release rate is governed by the synergistic effects of multiple contributing factors.

#### 3.4.3 Temperature-pH dual responsive cryogels

**Fig. 7:**
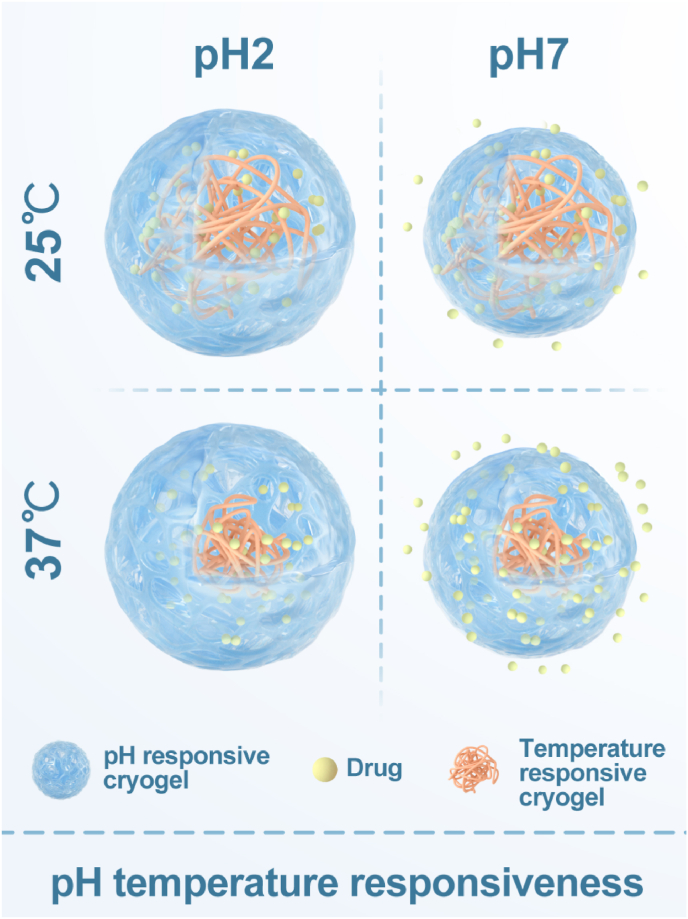
Schematic diagram of drug release of PH-temperature dual-responsive cryogels. (Different monomers of cryogels exhibit distinct trends in swelling rates as temperature and pH vary. These observations are detailed in Chapter 3.2.1 for temperature-responsive cryogels and in Chapter 3.2.2 for pH-responsive cryogels, respectively. This schematic diagram uses LCST temperature-responsive cryogels and chitosan cryogels as illustrative examples.)

Synthetic cryogels can be engineered by integrating temperature-sensitive monomers or polymers with gel monomers that contain functional groups, enabling the development of temperature-pH dual-responsive cryogels for controlled drug release in two dimensions (Fig. 7). The varying binding modes that confer dual regulatory properties on gels result in structural and physical variations, which in turn produce subtle differences in drug release profiles. These differences offer opportunities for further exploration and optimization of drug delivery systems. Specifically, leveraging the temperature responsiveness of N-isopropylacrylamide and the pH responsiveness derived from the ionization property of -NH2 in chitosan molecules, Wang et al. successfully designed and characterized a temperature-pH dual-responsive hydrogel by grafting N-isopropylacrylamide copolymer onto chitosan. They also investigated how the combination of these two substances influenced the responsive properties [106]. Similar experiments were also conducted by Bao et al. [107]. Furthermore, Seetapan et al. carried out an investigation into the linear viscoelastic properties of temperature-pH bi-responsive hydrogels with identical compositions [108]. The drug release properties of one of the NIPAAm-chitosan cryogels were further investigated by Huang et al., and the results demonstrated that these dual responsive cryogels exhibited a controlled and sustained drug release profile. Additionally, it was observed that the drug release rate could be enhanced through enzymatic hydrolysis of chitosan or with the aid of hydrogen peroxide [109]. The findings of Lee and colleagues are consistent with similar conclusions [110]. Catalina et al. achieved localized controlled mucosal delivery of the antifungal drug voriconazole by constructing Chitosan-Graft-Poly(N-Isopropylacrylamide)/PVA cryogels [111].

### 3.5 Cryogels as cell delivery platforms

Cell delivery refers to the process of loading and delivering functional cells (such as stem cells, progenitor cells or differentiated cells) to target tissues or organs by using carriers with appropriate pore structure, mechanical strength and good biocompatibility, in order to achieve tissue repair or regeneration (cell proliferation and differentiation in the target organ/tissue, replacing or supplementing to rebuild its structure) or functional reconstruction (such as hormone secretion, antibody production). Currently, cell therapy has emerged as an indispensable component of contemporary medical treatments, playing a pivotal role in tissue regeneration, cancer therapy, immunotherapy, and other domains [112]. However, maintaining cellular activity and optimal biological functionality during the delivery process presents significant challenges in terms of the structural design, microenvironmental conditions, and biocompatibility of the delivery system. The pore size of traditional hydrogels is too small to effectively facilitate cell loading within the pores. However, the excellent biocompatibility of hydrogels enables cell embedding/in-situ gelation. Cell embedding/in-situ gelation depend on hydrogel degradation to provide space for cell proliferation and extracellular matrix secretion. Therefore, it is essential that the hydrogel material exhibits a controllable degradation rate and does not produce degradation byproducts with potential biotoxicity or disrupt local environmental homeostasis (for example: pH) during degradation. Moreover, most injectable in situ forming hydrogels are initially delivered as liquid precursors that subsequently solidify within the body [113,114]. Typically, the injected gel precursor solution forms a hydrogel through either chemical (e.g., Michael-type addition reactions, disulfide bond formation, click chemistry, radical polymerization) or physical (e.g., ionic interactions, hydrogen bonding, hydrophobic interactions) crosslinking mechanisms [115]. However, this approach is associated with several limitations, such as inadequate gelation time, formation of hydrogels with insufficient mechanical strength, potential toxicity, and an inability to adequately protect encapsulated biomolecules or cells in complex biological environments [49]. Furthermore, the liquid precursor solutions may leak into surrounding tissues or become diluted in bodily fluids, which can not only hinder proper hydrogel formation but also compromise the resulting gel's properties [13,116,117]. These limitations consequently hinder the broader application of hydrogels in cell delivery. As mentioned above, cryogels synthesized from natural or synthetic monomers or polymers exhibit remarkable biocompatibility. Furthermore, cryogels possess large mesh sizes and demonstrate exceptional inter-pore connectivity, facilitating the attenuation of stresses through reversible deformations during substance delivery and it can promote the transport of substances inside the mesh, promote the delivery of nutrients and the discharge of metabolic waste. Cryogels' versatile fabrication process accommodates a variety of materials, enabling synthesis from single or multiple components with subsequent modifications based on this flexibility. In cases where a single gel component fails to meet all cellular requirements for an effective delivery system, optimization can be achieved through the incorporation of multiple gel matrices or other active ingredient admixtures. Furthermore, the freezing process does not compromise their inherent biological properties [118]. The exceptional mechanical properties and injectability of their synthesized hyaluronic acid cryogels, identified by Rezaeeyazdi et al., were found to exhibit poor NIH-3T3 fibroblast adhesion. In contrast, gelatin cryogels presented weak mechanical properties yet remarkable biocompatibility and cell adhesion due to the presence of RGD sequences. Therefore, a blend of the two components was synthesized, yielding HA-co-Gelatin cryogels that capitalize on the beneficial properties of each polymer to elicit a synergistic effect [118]. A similar conclusion was reached by Burns et al. [12]. All these demonstrated the pivotal role of cryogels in immunocell therapy, stem cell therapy, and tissue regeneration [[119], [120], [121], [122], [123]]. The starPEG-heparin cryogel scaffold material designed by Aliperta et al., was functionalized with RGD sequences to enhance cell adhesion and the rate of cell loading. Genetically modified MSCs were attached to the scaffold, creating a stem cell factory capable of treating acute myeloid leukemia (AML). The MSCs loaded in the cryogels exhibited excellent adhesion, migration ability, and active biological function. In vivo and in vitro experiments have demonstrated that this system can continuously release fully humanized anti-CD33-*anti*-CD3 bispecific antibody (bsAb), efficiently redirecting CD3^+^ T lymphocytes to CD33^+^ AML mother cells for exerting anti-tumor effects [124]. In a study conducted by Kim et al., heparin-modified injectable gelatin cryogels exhibited remarkable cell adhesion and survival rates both before and after injection, demonstrating their efficacy in promoting blood vessel regeneration when loaded with fibroblasts and endothelial growth factor [77]. Béduer et al. observed that cryogels composed of sodium alginate-carboxymethylcellulose and loaded with metaplastic neurons not only facilitate enhanced cell adhesion but also effectively safeguard the integrity of the neural network during reinjection [125]. In the process of producing cell loaded cryogels, the loading of cells into cryogels is performed after cross-linking, as the freezing process in cryogels preparation may potentially compromise the viability and biological functions of cells, further necessitating the production, purification, and sterilization of cryogels prior to cell loading [6]. With the advancement of 3D printing technology, cryogels have also found applications in the field of 3D bioprinting with cell incorporation. Cells are deposited onto the cryogels substrate through a printing nozzle, adhering and proliferating to actively participate in the formation and functionality of tissue- and organ-like structures [49,126,127]. Even minor traumas can result in bleeding, dysfunction, and other severe consequences for delicate or fragile organs or tissues such as the brain and liver. To further enhance the minimally invasive nature of treatments, some researchers are currently exploring the development of injectable micro-cryogels for cell therapy [128]. Newland et al. fabricated PEG-heparin micro-cryogels, approximately 300 μm in diameter, which demonstrated smooth injectability through a 27G needle and preserved the structural integrity and high viability of the neuronal cells they carried [129]. Another advantage of such micro-cryogels is their ability to be more uniformly distributed within the tissue post-injection, enabling them to effectively fill large or irregular tissue defects [130]. However, enhanced dispersibility and reduced size may increase the posibility of leakage beyond the target tissue [6]. Alisa et al. further highlighted that cryogel is not only suitable for cell loading but can also directly freeze the loaded cells without causing damage during the freezing process due to its rapid water absorption capability. The recovery of human mesenchymal stem cell (HMSC) activity was observed after the alginate gel-protein lectins containing HMSC were resuscitated from cryogel preservation [131].

### 3.6 Cryogels facilitate the co-delivery of cells and molecules/drugs

Tissue regeneration and biotherapy often necessitate the concurrent use of both cells and molecules/drugs. As previously discussed, cryogels can regulate the release of molecules/drugs by integrating various intelligent regulatory mechanisms, while enabling cell loading, protection, and cryopreservation through their unique structural features. As a drug delivery system, cryogels modulate drug release kinetics. Compared to the surrounding solution, drugs are preferentially released into the macropores and reach equilibrium more rapidly, meaning that when cryogels serve as dual carriers for both cells and molecules/drugs, cells migrating into or colonizing the macropores of cryogels are exposed earlier to higher concentrations of the molecules/drugs. The application of cryogels for the co-delivery of cells and factors not only enhances the induction effects of molecules/drugs on cells within cryogels but also achieves controlled release and protective delivery of molecules/drugs to cells. Kim et al. encapsulated VEGF and NIH-3T3 fibroblasts within heparin-gelatin cryogels and evaluated the controlled release of VEGF and the protective effect of cryogels on NIH- [10]3T3 cells during injection. Furthermore, in vivo ischemic hind limb mouse models demonstrated that VEGF/NIH-3T3/cryogels exhibited significantly faster blood flow recovery and lower incidence of limb necrosis compared with cryogels loaded solely with VEGF or with NIH-3T3 fibroblasts. This system also increased CD31 expression and enhanced neovascularization [77]. Sievers and colleagues developed injectable Starpeg-Heparin Cryogels encapsulating NGF and rat pheochromocytoma (PC-12) cells to examine the impact of varying concentrations of NGF on PC-12 neuronal differentiation in vitro. Their studies demonstrated that cryogels loaded with NGF and PC-12 cells significantly enhance nerve cell axon growth. Most importantly, the results clearly demonstrate that the differentiation response of PC-12 cells cultured within ready-to-use cryogels was comparable to that of freshly loaded cryogels [10]. These findings suggest that the Starpeg-Heparin cryogel system not only exhibits excellent usability but also effectively mimics neurogenic niches [132,133], potentially serving as a delivery vehicle for growth factor or cell therapy in the context of Parkinson's disease [10,68,134].

## 4 The latest applications of cryogels as DDS in the biomedical field

Cryogels are widely applied as a platform for cell/protein/drug delivery and engineering of different tissues [45]. We have previously discussed the preparation methods, structural characteristics, and delivery principles of cryogels. In this chapter, we present a summary of the application potential of cryogels as delivery carriers in Table 2 for clarity and conciseness.

**Table 2:** The application of cryogels as delivery system.

Cargo		Cryogels	Delivery principle	Application	Reference
molecules/drugs	GM-CSF, Flt3L, IL-15, CCL20	laponite modified alginate cryogel	affinity regulation depending on the electrostatic attraction	Protein molecular controlled-release and delivery	[67]
	outer layer: VEGF; inner layer: BMP-4	outer layer: gelatin/heparin cryogel; inner layer: gelatin/chitosan cryogel	The sequential release controlled by a dual-layer structure; affinity regulation depending on the electrostatic attraction	Vascularized bone regeneration	[69]
	IL-10; TGF-β; VEGF; FGF	Chitosan cryogel disk	Mesh size control	Control inflammation and accelerate wound healing	[78]
	bovine serum albumin, BSA	Zwitterionic Croigels	affinity regulation depending on the electrostatic attraction	The protein loading rate can reach up to 80 %, with sustained protein release lasting for a period of up to four months	[79]
	microRNA-146A + reactive oxygen species-scavenging cerium oxide nanoparticles (CNPs)	Zwitterionic Croigels	affinity regulation depending on the electrostatic attraction	Diabetes chronic wound healing	[135]
Cell	C2C12 Cell	Cylindrical HEMA Agarose cryogel/Geltain cryogel	High toughness/mechanical protection	Transportation and cryopreservation of C2C12 myoblast cell line	[136]
	PNIPAAm hepatic spheroids	Cylindrical PEG-Alginate-Gelatin(PAG)	Simulates natural cell polarity and enhances cell activity and proliferation	Development of artificial liver tissue in vitro	[137]
	Mature dopaminergic neuron: the LUHMES cell line	Cylindrical Carboxymethyl cellulose (CMC) cryogel	Minimally invasive delivery; Improve cell adhesion and survival rate	Mature nerve tissue transplantation	[138]
	hematopoietic stem and progenitor cell (HSPC)	collagen-coated carboxymethylcellulose cryogel bulk	Maintain stem cell activity and differentiation potential	injectable hematopoietic niche	[139]
	chondrocyte	gelatin/glucosamine cryogel microbeads	Large mesh size and injectability	cartilage tissue engineering	[140]
	pancreatic islets and mesechymalmscymal strolomal Cillomal (MSC)	starPEG-heparin cryogel	Adhesion peptide modification	Intrahepatic transplantation of allogeneic pancreatic islets	[141]
molecules/drugs and cells	GDNF/NGF + MCS/PC12	StarPEG- heparin cryogel	High biocompatibility; Injectability; affinity regulation depending on the electrostatic attraction	Treat Anoikis; treatment of neurological diseases such as Parkinson's disease	[134]
	VEGF/NIH-3T3 Fibroblasts	Gelatin-Heparin Cryogels	affinity regulation depending on the electrostatic attraction	Hindlimb ischemic disease treating and revascularization	[77]
	Human dermal fibroblasts/Mitoxantronec	Alginate cryogels modified with RGD and doped with Fe_3_O_4_	Magnetic field-mediated regulation of cell and drug release	Cell and drug controlled-release	[142]
	Simavastatin and Stem cells from human exfoliated deciduous teeth	GelMA cryogel microspheres	PLGA nanoparticles engineered for controlled drug release	Vascularized pulp regeneration	[143]

## 5 Biomimetic design of cryogels

**Fig. 8:**
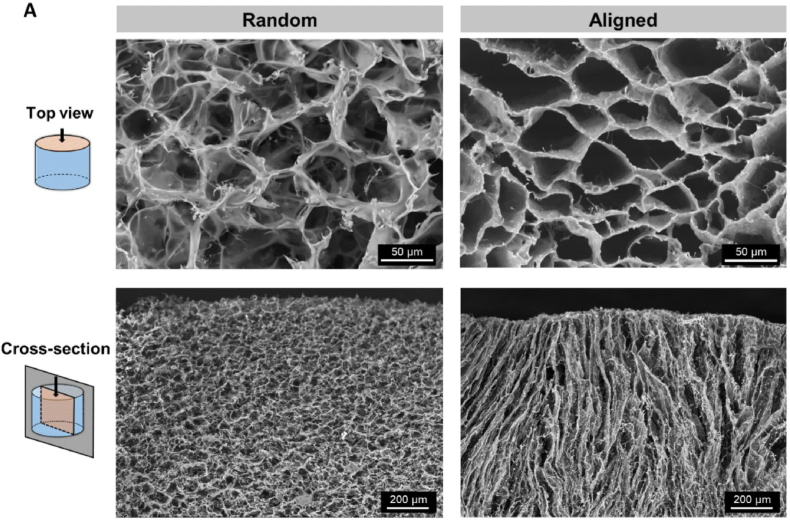
Random and aligned AMMA cryogels. Represented with the permission from Sousa et al. (2024), Biomaterials Science [147].

**Fig. 9:**
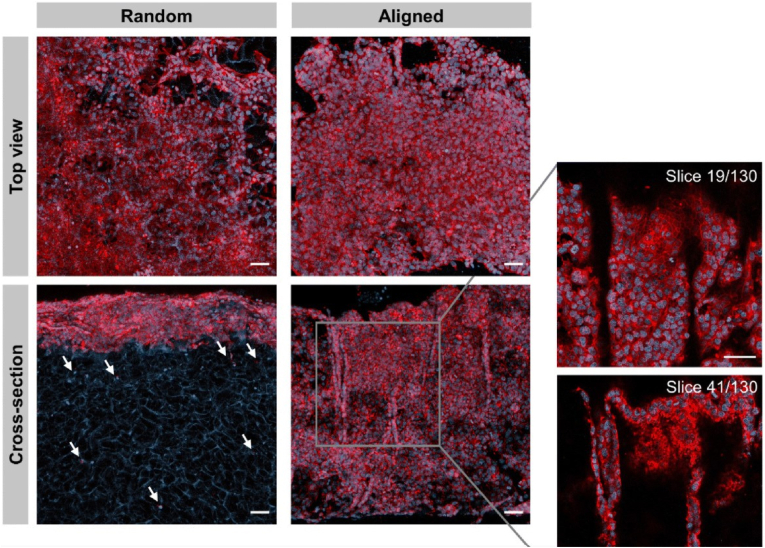
Proliferation of NSC cultures on random and aligned cryogels. Phalloidin (red) and DAPI (blue) staining reveals the morphology and distribution of NSCs cultured on random and aligned cryogels after 7 days. The white arrows highlight the sparse distribution of cells within the random cryogels. Scale bars = 100 μm. Represented with the permission from Sousa et al. (2024), Biomaterials Science [147]. (For interpretation of the references to color in this figure legend, the reader is referred to the Web version of this article.)

**Fig. 10:**
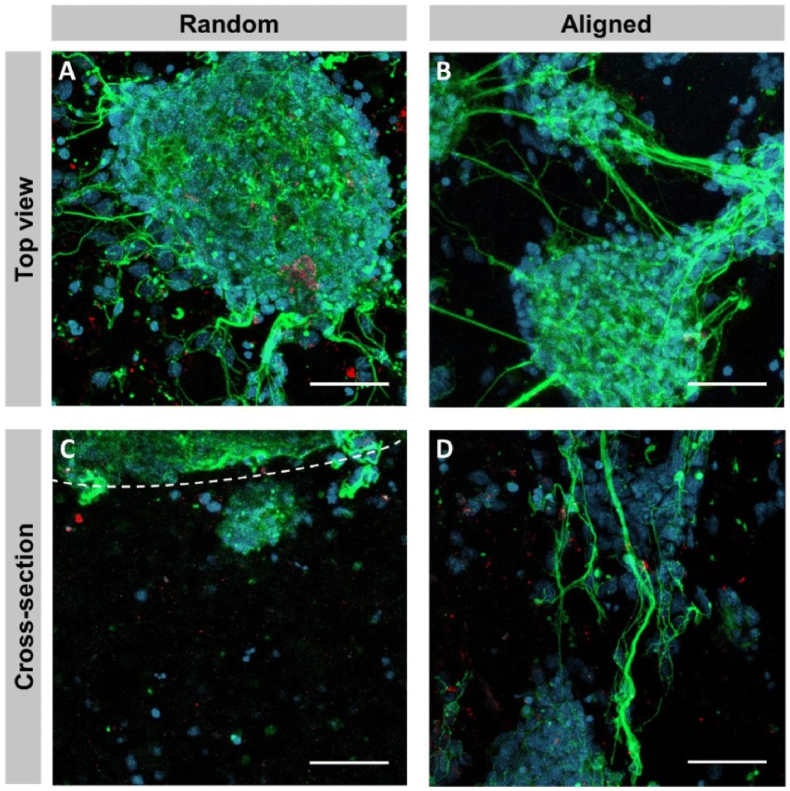
Differentiation of NSCs cultured on random and aligned cryogels. A and B: On the surface of the cryogels, neurons were predominantly organized into clusters with extending neurites. C: Cell infiltration within the random cryogels was minimal, whereas D: cell clusters inside the aligned cryogels extended neurites along the longitudinal pores. Represented with the permission from Sousa et al. (2024), Biomaterials Science [147].

In recent years, researchers have been actively investigating the immense potential of cryogels in the field of bionics, gradually shifting their focus from synthetic compounds to naturally occurring polysaccharides [144,145], macromolecular proteins [146], and even unconventional sources such as pollen [149] and amniotic membrane [147]. The integration of bionics and cryogels is not only reflected in the selection of materials but also in the design of the microstructure of cryogels. Some scholars have altered the distribution direction of the mesh of cryogels through controlled freezing techniques, creating structures that are more in line with the original tissue arrangement of organisms or more conducive to cell migration and the exertion of biological functions [147]. Specifically, Deng et al. fabricated cryogels utilizing the distinctive hierarchical structure of natural non-allergenic pollen, and the cryogels exhibited fluid-induced shape memory functionality within an impressive 2-s timeframe as well as exceptional hemostatic efficacy in animal experimentation [148]. Sousa et al. highlighted the pivotal role of physical cues in guiding neural cell growth and underscored their therapeutic potential [149]. They contended that the organized arrangement of cells and the extracellular matrix enables the efficient and uninterrupted transmission of electrical signals along axons in the spinal cord and peripheral nerves [147]. They utilized a vertical, unidirectional temperature gradient to produce anisotropic, methacryloyl amniotic membrane (AMMA) cryogels which has aligned mesh walls. In contrast, traditional cryogels exhibit random mesh walls [150] (Fig. 8). Cross-sectional analysis demonstrated that random cryogels exhibited restricted cellular infiltration, with the majority of cells clustering at the surface boundary. By day 7, only 11 ± 1 % of the cross-sectional area was occupied by cells and the viability ranged between 74 ± 2 % and 78 ± 1 %. In contrast, in aligned cryogels, cells were detectable within the scaffold as early as day 1 and progressively increased in density over time. The cell-covered area increased from 10 ± 5% to 28 ± 3% on day 1 and 44 ± 5% on days 4 and 7, with the viability ranging between 79 ± 12 % and 94 ± 1 % [147](Fig. 9). Additionlly aligned cryogels demonstrated better axonal growth (Fig. 10). Biological tissues typically possess regular fine structural arrangements. These disparities in structure can significantly alter cellular behavior. In the realm of structural bionics, academic focus has been extended beyond biological tissues. For instance, inspired by the natural lotus leaf structure, Kanmaz et al. designed a gel-nanofiber hybrid cryogel that fulfilled tissue engineering requirements [151]. In terms of cryogels' application, it extended beyond traditional tissue engineering or material delivery, and encompassed the integration of cryogels with electricity and magnetism to enhance materials control and regulation [152,153]. Furthermore, cryogels can be employed for in vivo homeostasis regulation or immune microenvironment modulation, allowing for precise immunotherapy and investigation into strategies for evading tumor immune evasion [[154], [155], [156]].

## 6 Conclusion and outlook

Cryogels, created through cryostructuring and using solvents as porogenic agents, have gained attention for their distinctive structural features: large mesh size, high porosity, interconnectivity, injectability, shape-memory properties, superior biocompatibility, and notable biological performance. The versatility of the production method, applicable to a wide range of gel monomers and polymers, enhances its practicality and convenience for various applications. Cryogels have seen widespread application in tissue engineering over the past decade, with uses in promoting wound healing, regenerating bone and cartilage, and facilitating nerve regeneration and angiogenesis as scaffold materials. They also enable targeted drug delivery, acting as substrates for 3D-printed organoids and tissues, and are integral to processes of biological separation, purification, and identification, as well as playing a substantial role in biosensing. This paper presents an overview of the properties, characteristics, and synthesis process of cryogels, with a specific focus on their current status and role as substance delivery vehicles for the controlled release of drugs and biomolecules. In terms of controlled delivery the freezing process of cryogels can increase mesh size and porosity, potentially leading to an initial burst release. However, post-freezing cryogels show improved strength and enhanced mechanical properties over Hydrogels, which is conducive to controlled drug release. However, the release rate is influenced not only by the diffusion resistance of the drug/molecule or the degradation rate of the delivery carrier but also by the release kinetics, which has been investigated by only a few scholars. By reviewing relevant literature, we elucidate how cryogels as delivery carriers alter release kinetics. Specifically, drugs/molecules loaded onto cryogels first achieve concentration equilibrium (local release) within the large pores of the cryogels. Subsequently, these drugs are released into the surrounding solution at a slow rate in the absence of agitation, eventually achieving global release equilibrium over an extended period. This shift in release dynamics represents an advantage of cryogels over traditional hydrogels as delivery vehicles. Furthermore, various modulations commonly employed in hydrogel systems to regulate controlled release—such as affinity regulation, enzyme responsiveness, pH sensitivity, temperature responsiveness, and other stimulus-responsive mechanisms—can similarly be applied to cryogel systems to enhance control over the delivery process. For cell delivery applications, cryogels facilitate cell adhesion on their surfaces while enabling cell loading, proliferation, and migration within their porous structures. The high mechanical toughness of cryogels allows them to be injectable, providing protection for cells during injection, which is crucial for minimally invasive cell delivery, particularly in regenerative medicine.

Of course, cryogels still hold significant potential for further development in the field of drug/molecular/cellular delivery. For instance, utilizing natural substances (such as natural polysaccharides) as materials for cryogel preparation; enhancing control over cryogel mesh morphology (including structure, orientation, and anisotropy); and employing the cryogel system to modulate release kinetics for co-delivery of cells and drugs/molecules can facilitate exposing cells to higher concentrations of drugs/factors compared to peripheral solutions, thereby enabling more effective regulation of cell behavior. Additionally, in the context of cell delivery, it is crucial to investigate the effects of cryogel property modifications and compositional alterations on cell differentiation and functionality. Furthermore, there is a need to accelerate the transition of these findings into clinical practice.

## CRediT authorship contribution statement

**Mucong Li:** Writing – review & editing, Writing – original draft, Software, Project administration, Investigation, Conceptualization. **Xuyan Wei:** Writing – review & editing, Data curation, Conceptualization. **Jiaqian You:** Writing – review & editing, Data curation, Conceptualization. **Jian Feng:** Writing – review & editing, Software. **Xiuyu Liu:** Writing – review & editing, Software. **Jing Zhou:** Writing – review & editing, Software. **Qianju Wu:** Investigation, Data curation. **Yidi Zhang:** Software, Investigation. **Yanmin Zhou:** Writing – review & editing, Software, Investigation, Funding acquisition, Data curation.

## Declaration of competing interest

The authors declare that they have no known competing financial interests or personal relationships that could have appeared to influence the work reported in this paper.

## Data Availability

No data was used for the research described in the article.
